# Honey compositional convergence and the parallel domestication of social bees

**DOI:** 10.1038/s41598-022-23310-w

**Published:** 2022-10-31

**Authors:** Pierre Noiset, Nathalie Cabirol, Marcelo Rojas-Oropeza, Natapot Warrit, Kiatoko Nkoba, Nicolas J. Vereecken

**Affiliations:** 1 Agroecology Lab, Université Libre de Bruxelles (ULB), Boulevard du Triomphe CP 264/02, 1050 Brussels, Belgium; 2 Functional Soil Microbial Ecology and Environmental Protection Group − Department of Ecology and Natural Resources, Facultad de Ciencias, Universidad Nacional Autonoma de Mexico, 04510 México City, México; 3 Center of Excellence in Entomology and Department of Biology, Faculty of Science, Chulalongkorn University, Bangkok, Thailand; 4 International Centre of Insect Physiology and Ecology, P.O. Box 30772-00100, Nairobi, Kenya

**Keywords:** Entomology, Chemical ecology, Evolutionary ecology

## Abstract

Honey collection evolved from simple honey hunting to the parallel and independent domestication of different species of bees in various parts of the world. In this study, we investigate the extent to which the composition of *Apis* and stingless bee honeys has been a driver in the selection of different bee species for domestication in Mesoamerica (Mexico) and Asia (Thailand) using a sampling design that combines peak honey profiling by H1 NMR spectroscopy with the collection of honeys from domesticated and undomesticated bee species. Our results show that, independently of the region of the world considered, domesticated stingless bees produce honey whose compositional profiles differ from those of the non-domesticated species and exhibit more similarities towards honeys produced by the domesticated *Apis* species*.* Our results provide evidence for the first time that the search for natural sweeteners in the environment by our ancestors led to the parallel and independent domestication of social bees producing honeys with similar compositional profiles.

## Introduction

Honey has long been a key source of carbohydrates in the human diet throughout the pre-industrial times, as it was one of the only concentrated form of sugars directly obtained from the environment^1^. Archaeological evidence showed that Stone Age people already harvested bee products, including honey^2^. Honey hunting or the gathering of honey directly from wild bee colonies is an ancient human activity still practiced to the present day. If the traditional methods of honey hunting in some areas ensure the sustainability of wild colonies by leaving the brood intact, the increasing population and pressures on wild resources often results in destructive practices leading to the decline of bee colonies and habitats in addition to poor quality products^3^. This shortcoming led human societies to develop innovative animal husbandry and management practices through the application of their knowledge of behavioural ecology which led to the parallel and independent domestication of different species of honey-producing social bees in different parts of the world^4^. While beekeeping had developed with the well-known *Apis mellifera* (L., 1758) in Europe, Africa and the Middle East, the prevalent species in Asia was *A. cerana* (Fabricius, 1793) whose breeding started later in China, around 200 AD^5^. In Mesoamerica, beekeeping emerged within the Mayan civilization around 1750–2300 BP with another tribe of eusocial bees, stingless bees (Apidae, Subfamily *Meliponinae*).

Stingless bees, or meliponines, are honey-producing bees that inhabit tropical and subtropical environments all over the world^6^. There are about 550 described species, most of which are found in the Neotropics where meliponiculture (i.e., beekeeping with stingless bees) developed during pre-Columbian times, particularly in the Yucatan peninsula and the Mayan civilization^7^. Historically, the rearing of *Melipona beecheii* (Bennet, 1831) was even described as an important activity generating income through the trade of cerumen and honey used for food consumption or for their claimed medicinal properties^8^. The arrival of European conquistadors during the sixteenth century marked the beginning of the decline of meliponiculture driven by (i) the introduction and promotion of apiculture, which progressively replaced stingless beekeeping as it produces comparatively more honey, and (ii) the increasingly widespread cultivation of sugarcane (*Saccharum officinarum*), which rapidly provided yet another important source of sweetener^9–12^. The apparent decline of stingless bee populations is also likely to have been reinforced by contemporary anthropogenic environmental change, including habitat loss and deforestation particularly important in the Neotropics^13^, climate and land use change, and the introduction of exotic species^14^. By contrast, beekeeping with *A. mellifera* in Mesoamerica has grown significantly; Mexico is currently the 6th largest honey producing country in the world^15^.

In parallel, meliponiculture is also gaining much interest in different regions of Asia, especially in Malaysia and Thailand, where beekeeping using *Apis* species is the norm, and stingless bee honeys are considered a source of additional income in rural areas where their honey is sold as a non-timber forest product with claimed medicinal properties. Indeed, the apparent medicinal and nutritional virtues of their honeys^16,17^, their comparatively low-cost management^18^, and their potential for the pollination of diverse insect-dependent crops^19–23^ make meliponiculture a powerful leverage tool for rural development and sustainable resource use^24,25^ alongside traditional beekeeping using *Apis* species in Asia.

In this study, and in line with previous investigations on the “domestication syndrome” documented in crops^26,27^ and animals^28,29^ but less frequently on insects^30^, we aimed to investigate how beekeeping using different bee species in different parts of the world was driven by the quest for similar traits in the bee species targeted by domestication. Our rationale is that the desire for sweetness or a “sugar fix” is biologically hardwired in human societies, making it a universal trait shared by humans that evolved in times when sources of carbohydrates were scarce in the natural environment^31^. We hypothesize that humans in different regions of the world independently and preferentially domesticated species of bees in the genus *Apis* and the *Meliponinae* subfamily that produced honeys characterized by similar physical and chemical characteristics (or “sensory adaptive peaks”) among the wider “compositional landscape” of available honeys produced by native social bees. Specifically, we used H^1^ NMR spectroscopy to investigate the physico-chemical profile of honey samples produced by *Apis* and stingless bee species in Mexico and Thailand (two countries where beekeeping and meliponiculture coexist, but in different historical contexts introduced above), including domesticated and non-domesticated species. Our goal was (i) to compare the honey profiles of domesticated species with those of non-domesticated bee species in each country, and (ii) to investigate potential similarities between honeys produced by domesticated species in each country in different regions of the world with significantly contrasting floras.

## Results

### Honey samples classification

We collected 60 honey samples in Thailand and 31 samples in Mexico which were analysed by H^1^-NMR spectroscopy. To compare honeys characteristics and the domestication state of the bee species, stingless and honey bee samples were classified a priori in three categories: (i) Apiculture (*Apis* spp.) (n = 26), (ii) Meliponiculture (*Melipona* and *Scaptotrigona* spp. in Mexico^8^, and *Tetragonula* sp. in Thailand^32^) (n = 37), and (iii) honeys from non-domesticated stingless bee species (n = 38).

### Multivariate analysis of honey composition and the parallel domestication of social bees

**Figure 1:**
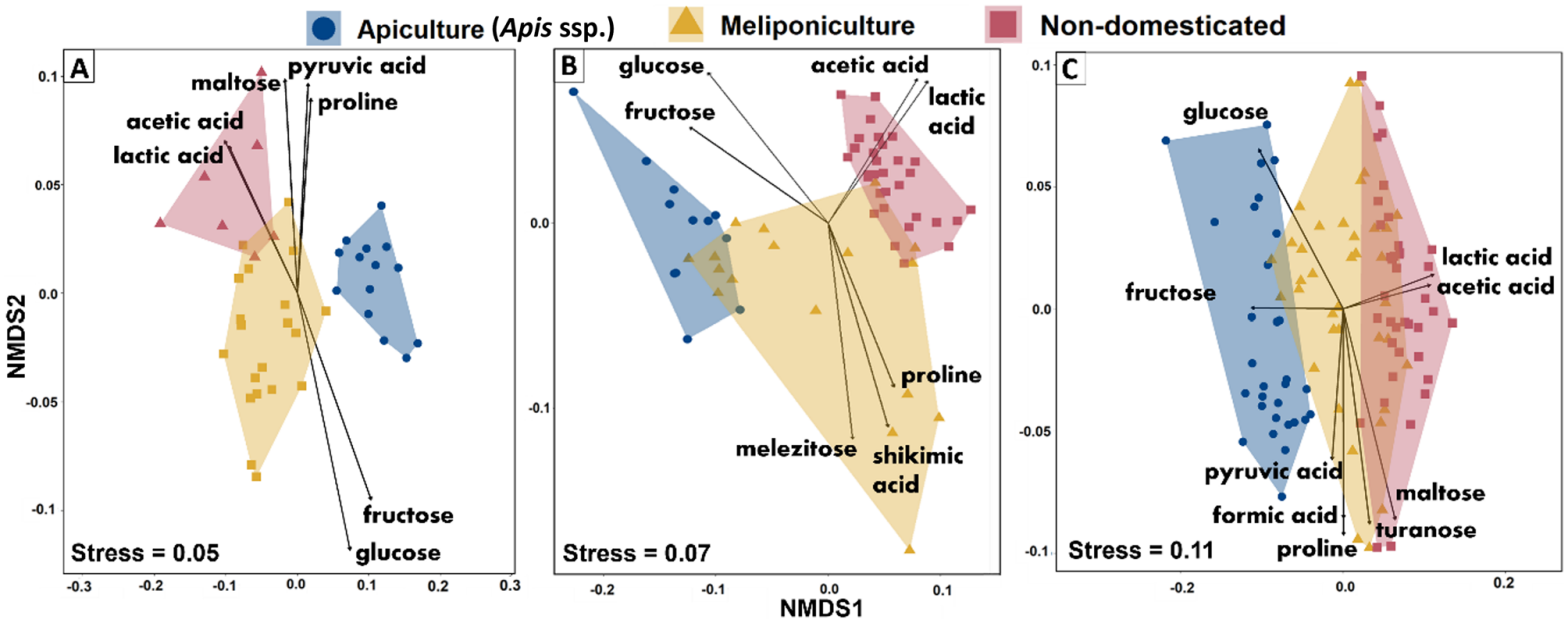
Non-linear Multidimensional Scaling (NMDS) ordination plots showing the differentiation of honey samples grouped into three convex hulls according to the domestication state of the bee species comprising samples from Mexico (**a**), Thailand (**b**) and from both countries (**c**). The results show stingless bee honeys were divided in two discrete groups with little to no overlap between domesticated and non-domesticated species. Honeys produced by domesticated stingless bee species consistently exhibit physicochemical profiles more similar to those of *Apis* honeys, characterized by a sweeter profile and lower fermentation marker level, compared to those of non-domesticated stingless species.

The analysis of similarities and the PERMANOVA performed on all the quantified variables (Supplementary Table S1) showed significant differences between the three groups in both countries (Mexico, *p*-value = 9.99 × 10^–5^, df = 2, R^2^ = 0.248; Thailand, *p*-value = 0.001, d*f* = 2, *R* = 0.6597). NMDS plot (Fig. 1A and B, see also Supplementary Figure S2) illustrated these differences, showing a different pattern in datasets from each country. In Mexico, stingless bee honeys differ from *Apis* species and formed two discrete clusters with little overlap between domesticated and non-domesticated species. In Thailand, domesticated stingless bee honeys formed a discrete cluster with a more variable composition compared to the results from Mexico and the cluster of domesticated stingless bee honeys overlapped with both *Apis* spp. and non-domesticated stingless bees. Major fermentation markers (acetic and lactic acid) and sugars (fructose and glucose) were recorded in all honeys irrespective of their country/region of the world, and they were the most relevant compounds to discriminate honeys of the three groups of species. We also performed PERMANOVA on the whole dataset (Mexico + Thailand) and found consistent patterns of differentiation among the three groups of samples, regardless of their geographical origin (*p*-value = 9.99 × 10^–5^, d*f* = 2, R^2^ = 0.264). As shown on the NMDS plot (Fig. 1C) and in line with the country-specific results described above, domesticated stingless bees produce honeys whose profiles tend to be more similar to those of *Apis* species, with higher fructose and lower fermentation markers levels than non-domesticated species honeys. Honeys from domesticated species (*Apis* spp. and stingless bees) were also associated to malic acid (stat = 0.795, *p*-value = 0.005) and methylglyoxal (stat = 0.626, *p*-value = 0.005).

## Discussion

In our study, we provide evidence for differentiation in honey physico-chemical characteristics between domesticated and non-domesticated species of stingless bees. Domesticated stingless bees produce honeys with a compositional profile similar to those produced by honey bee (*Apis*) species regardless of their geographic origin and even though our samples were collected at localities characterized by different climates and contrasting floras, namely the environments of Thailand in Asia and Mexico in Mesoamerica.

A first explanation for the patterns observed could be that they are the direct outcome of the domestication process targeting honey-producing bee species. Just as it has been demonstrated for crops and animals^27,29^, the selection of traits of interest in honey by humans, driven by the same needs and sensory biases, could indeed lead to evolutionary parallelism, at least theoretically. Although there is evidence that advanced techniques in artificial selection and genomic applications can theoretically lead to persistent behavioural changes with a strong genetic basis in *Apis* species (e.g. in hygienic behavior (HB)^33^ nest defense or aggressiveness^34^, foraging or nest defense^35^), we found no evidence for the impact of bee domestication on honey properties using controlled experimental design. Also, because the historical and contemporary techniques of meliponiculture are still largely elementary, consisting primarily in hosting wild stingless bee colonies in man-made wooden structures and in dividing colonies without a genetic control on the domesticated lines per se, we think that the “domestication scenario” is unlikely to explain the compositional patterns of honeys observed and characterized in this study.

Here, we favour an alternative hypothesis of “double sensory adaptation” driven first by a preference of bees for certain types of floral nectars, and second by a preference of humans for certain patterns of sugars in the honeys produced by different species of bees. The first “sensory adaptation” is based on the fact that bees play a selective role in shaping nectar characteristics which tend to be homogeneous for plants visited by the same pollinator group; the “pollination syndromes”^36,37^. Previous studies shown that bee-pollinated plants usually produce low nectar volumes associated with high sugar levels, while opposite pattern is observed in plants pollinated by birds, bats and lepidopterans^38,39^. This nectar composition adapted to bee preferences, or “sensory adaptation”, and its “variation on a theme”, will in turn delineate the compositional landscape of honeys. The secondary “sensory adaptation” is based on the hypothesis that human societies searching for sources of sugars preferentially engaged in meliponiculture using stingless bee species targeted both for their production of sweeter honey and their adaptation potential to man-made hives and beekeeping techniques^8^. Altough, this hypothesis does not fully account for the smaller overlap in honey composition between domesticated stingless bees and *Apis* in Mexico (Fig. 1A) compared to what is observed in Thailand (Fig. 1B). We suggest that the “sensory adaptation” hypothesis is the most likely scenario explaining the patterns observed. Other local factors such as the species-specific patterns of biochemical conversion of floral nectars into honey by bees in the phylogenetically-distant genera *Melipona* and *Scaptotrigona*^40^, leading to the observed country-specific patterns of honey composition among groups of bees (Fig. 1A,B). Likewise, the resource competition/partitioning/limitation might differ between the two regions investigated, leading stingless bees and honey bees to forage on different plants with different nectar profiles. These ecological and evolutionary mechanisms are not mutually exclusive, but they cannot be untangled in this study and deserve further examination with controlled experiments.

As illustrated by our results, a higher level of fructose, the sweetest natural carbohydrate^41^ and more broadly higher level of sugars must have been highly feature of honey as a source of energy and one of the only concentrated natural sweeteners available in the environment. Fructose is also a key compound for human consumption as it takes part in the hypoglycemic action of honey^42^ and its high sugar levels, combined with low fermentation rates and antioxidant properties, also contribute to the use of honey as food preservative. The higher level of methylglyoxal and malic acid observed in honeys produced by some domesticated stingless bee species could also indicate a greater therapeutic potential. Methyglyoxal contributes to the non-peroxide antibacterial activity and is a key compound of the famous manuka honey^43^. Malic acid, on the other hand, contributes to the antioxidant activity of honey trough metal chelation together with other organic acids^44^. These medicinal properties could be another driver for the selection some bee species over others. Indeed, *Apis* and stingless bee honeys from domesticated species are frequently used in traditional medicine with varying levels of success in Thailand and in Mexico to treat various diseases from skin infections to fertility issues, to neurological disorders^17^.

Investigating ecological and evolutionary drivers affecting the properties of honeys produced by social bees remain a challenge due to the many potential sources of variation. While current research is mainly focusing on the identification of chemical markers specific to a floral origin or on the establishment of standards in the case of stingless bee honey^45–47^, investigations into stingless bee ecology should be encouraged throughout Africa and Asia, where their ecology and patterns of evolution are more poorly understood. There is a major gap between our knowledge of honey bees and stingless bees, especially in Asia and Africa^6,46,48^. Disentangling the roles of shared evolutionary history and environmental conditions on honey properties is also still hindered by the fact that phylogenetic relationships among stingless bees still remain unresolved for most genera^40,49^. Furthermore, our understanding of their foraging patterns and resource partitioning among sympatric species remains limited, as is the effect of the potential competition with managed honey bee colonies. Future research should aim to fill these important gaps to allow addressing the ecology and evolution of honey properties at all scales, from local, regional, continental to the global scale. Such fundamental research will also be pivotal for the establishment of scientifically-informed standards for stingless bees to promote the generation of income through nature-based solutions and non-timber forest products in rural communities.

## Methods

### Sample collection

We collected 60 honey samples in Thailand and 31 samples in Mexico. In Thailand, stingless bees honeys (*n* = 48) from seven species were collected from managed colonies in five locations (Supplementary Table S3) during Spring 2021. Two extraction methods described by Mokaya et al.^50^ were employed to harvest honey directly from the nest. *Apis cerana* (*n* = 12) honeys from 2 species were extracted from managed colonies by squeezing the honeycomb. In Mexico, stingless bee honeys (*n* = 27) from eight species were directly gathered from local meliponiculturists in five regions (Supplementary Table S1) during Spring 2020. A volume of 100 ml of honey was collected from each colony and placed in a sterilized container following the method described by Quezada-Euán^8^. The stingless bee species identity was provided by the meliponiculturists during honey harvest. Samples of *Apis mellifera* honeys (*n* = 4) were collected from hives managed by the same meliponiculturists.

After their collection in the field, honey bee samples were stored in a fridge (< 5 °C) while *Meliponinae* honeys were stored in a freezer to restrict fermentation due to their higher water content. Indeed, the nectar is less dehydrated by stingless bees than by honey bees, allowing the presence of microorganisms that accelerate the fermentation of honey^51^.

### Honey profiling by ^1^H-NMR spectroscopy

To characterize the compounds and properties of our honey samples, we used Nuclear Magnetic Resonance spectroscopy (hereafter NMR spectroscopy), a state-of-the-art analytical technique increasingly used alongside chemometrics statistical approaches for the qualitative and quantitative control of honeys^52^, as well as to assess the botanical origin of honeys and quantify their major constituting compounds^53–55^.

NMR spectroscopy was carried out on all 91 samples described above at the laboratories of Quality Services International GmbH (QSI, Bremen, Germany). In short, sample preparation method for honey was adapted from Bruker Biospin GmbH (Rheinstetten, Germany). The homogenized honey samples (5 g) were solved in 17.5 ml NMR-buffer (KH_2_PO_4_, Merck; NaN_3_, Fluka; H_2_O) by shaking the samples for 30 min. Their pH was adjusted to 3.1 using an auto titrator and HCl 1 M (Chemsolute®). To reach 900 μL of each sample, 100 μL of internal standard have been added. The samples have been centrifuged for 15 min at 14,000 rpm and 600 μL of supernatant was ultimately transferred into a 5 mm NMR-tube (Deutero) for direct measurement.

All measurements were performed on a Bruker Avance III HD 400 MHz equipped with a 5 mm PA BBI 400SI H-BB-D-05 Z probe head. ^1^H-NMR-spectra were acquired at 300.1 K and calibrated using TSP as reference at 0.0 ppm. Tuning and matching, locking, shimming and pulse calibration was done with full automation. Measurement was done using NOESYPR1D as pulse sequence (1D Nuclear Overhauser effect spectroscopy with water pre-saturation), relaxation delay of 4 s, mixing time of 10 ms, number of scans of 32, acquisition time of 4 s, 64 k data points Time domain data, and a sweep width of 20 ppm. For the processing an exponential window function with line broadening of 0.3 Hz and zero filling, Fourier transform into 128 k frequency points, data processing using standard spectrometer software, baseline and phase correction with an automated processing program was used. The compounds were quantified by using the Honey-Profiling™ 2.0 routine (Bruker Biospin GmbH, Rheinstetten, Germany) by automatic integration of the specific peak areas calculated with an external standard using ERETIC function.

### Statistical analyses

Physicochemical data of honey samples of *Apis mellifera* (n = 10) from Mexico analysed according to the protocol described above at the QSI laboratories were pooled with our dataset; this allowed to reach a better balance between the number of honey bee and stingless bee samples analysed in this study.

All the statistical analyses presented here were performed in Rstudio^56^ for R^57^ using the *vegan* package (version 2.5–7)^58^. Our raw data have first been log transformed to limit the influence of higher values, and then standardized using the double Wisconsin transformation. Using this approach, each value in the “species *x* compounds” matrix is divided by its column maximum, and then divided by the row total, producing values between 0 and 1 that equalize emphasis among sample units and among species. Bray–Curtis dissimilarities have been computed on the transformed data to conduct multivariate analyses. We then performed an analysis of similarities (ANOSIM) or a Permutational Multivariate Analysis of Variance (PERMANOVA) when the group dispersions were heterogeneous to test whether there were significant differences among different groups of honey samples (e.g. honey bees *vs* stingless bees; honeys used a sweetener or consumed *vs* other honeys). These differences between groups of honey samples were illustrated trough Non-Metric Multidimensional Scaling (NMDS) plots using the *ggplot2* package (version 3.3.3)^59^ and a dendrogram (hierarchical clustering) using the *ggtree* package (version 3.4.1)^60^. Variable selection was done after performing a PCA and having retained the highly correlated variables to the principal components to obtain a reduced dataset (R^2^ > 0.8). NMDS analyses were performed on the reduced dataset to improve the representation. Indicator compound analysis was performed to identify the compounds associated with the different groups described above using the *indicspecies* package (Version 1.7.9)^61^.

## Supplementary Information

Supplementary Information.

## Data Availability

The data that support the findings of this study are available from the corresponding author upon reasonable request.
