# Supplementary material for: Honey compositional convergence and the parallel domestication of social bees
Source: Sci Rep. 2022 Oct 31;12:18280. doi: 10.1038/s41598-022-23310-w (PMC9622900; doi:10.1038/s41598-022-23310-w)
Supplement: Supplementary file 1 — Supplementary Information. [file 41598_2022_23310_MOESM1_ESM.docx]

| **Sugars** | | **Organic acid** | **Amino acids** | | **Fermentation markers** | | **Others** |
| --- | --- | --- | --- | --- | --- | --- | --- |
| Fructose | Melezitose | Citric acid | Alanine | Valine | 2,3-butanediol | Lactic acid | Dihydroxyacetone |
| Glucose | Maltotriose | Malic Acid | Aspartic acid | Tyrosine | 5-HMF | Formic acid | Methylglyoxal |
| Sucrose | Gentiobiose | Quinic acid | Glutamine | Phenylalanine | Acetic acid | Fumaric acid |  |
| Turanose | Raffinose | Shikimic acid | Leucine |  | Acetoin | Pyruvic acid |  |
| Maltose | Mannose | 3-phenyllactic acid | Proline |  | Ethanol | Succinic acid |  |

**
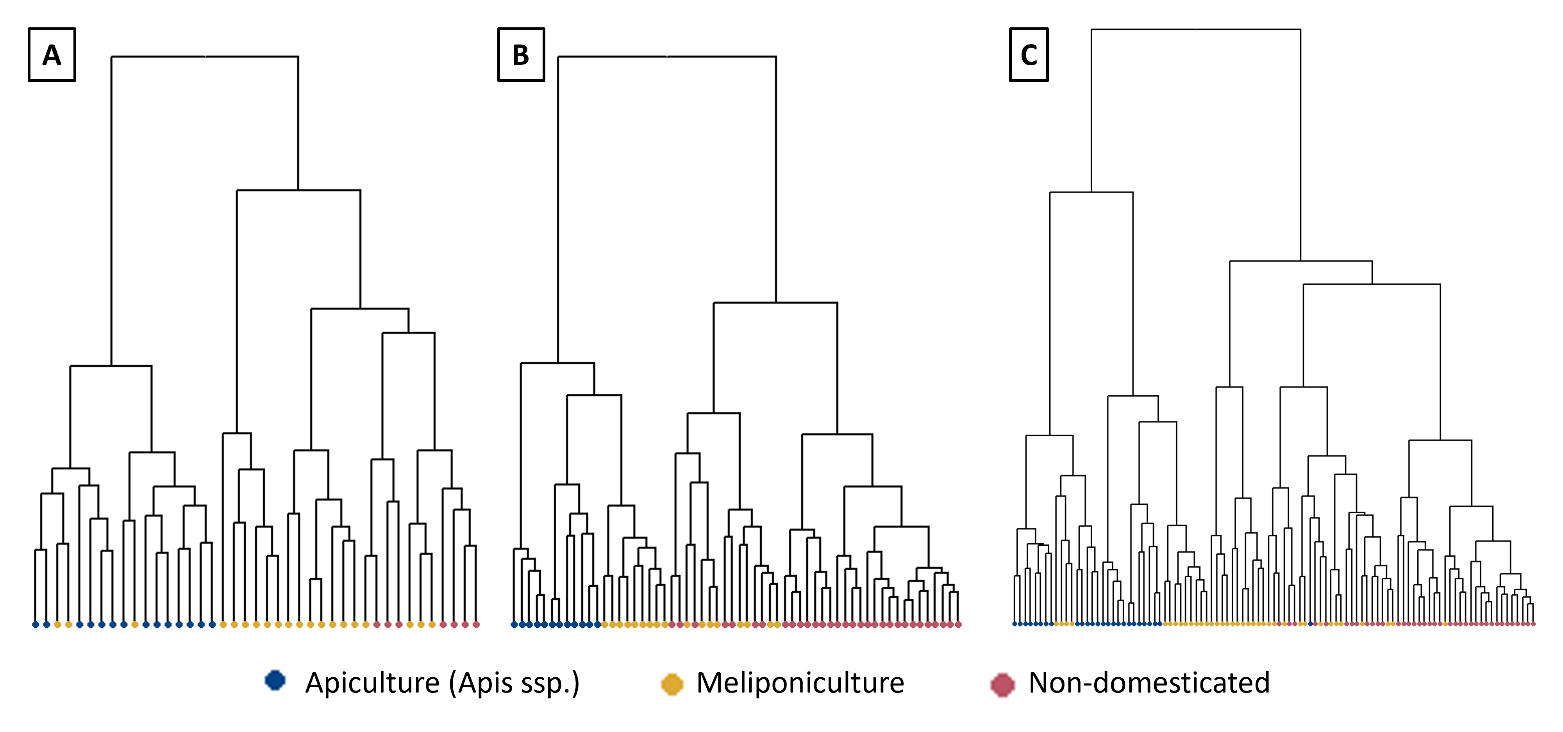
Supplementary Table S1** : Compounds identified and quantified by H1 NMR spectroscopy in each honey samples

**Supplementary Figure S2** : Dendogram of hierarchical clustering (Ward D2 method) illustrating the Bray-Curtis distances among each sample colored according to the domestication state of the bee species comprising samples from Mexico (a), Thailand (b) and from both countries (C).

| Country | Region | Latitude (°) | Longitude (°) | Honey samples | | |
| --- | --- | --- | --- | --- | --- | --- |
|  |  |  |  | *Apis* spp. | Meliponiculture | Non-domesticated |
| Thailand | Chanthaburi | 12.7655 | 102.193378 | 5 | 5 | 1 |
|  | Chiangmai | 19.7296333 | 99.1184694 | 0 | 8 | 2 |
|  | Patthalung | 7.8062167 | 100.017169 | 5 | 3 | 25 |
|  | Ratchaburi | 13.590325 | 99.5072028 | 2 | 0 | 0 |
|  | Saraburi | 14.5225278 | 101.024942 | 0 | 1 | 3 |
| Mexico | Altotonga | 19.783810 | -97.111098 | 1 | 1 | 2 |
|  | Atzalán | 19.875556 | -97.012222 | 0 | 2 | 0 |
|  | Conkal | 21.075145 | -89.520158 | 0 | 1 | 0 |
|  | Cuanixtepec | 20.134935 | -97.725639 | 0 | 2 | 1 |
|  | Cuetzalan | 20.026002 | -97.486833 | 0 | 4 | 0 |
|  | Huehuetán | 14.977895 | -92.333857 | 1 | 0 | 0 |
|  | Huixtla | 15.230425 | -92.467388 | 0 | 2 | 0 |
|  | Mérida | 20.977222 | -89.654500 | 0 | 3 | 2 |
|  | Pucnachén | 20.366827 | -90.219171 | 0 | 1 | 0 |
|  | Tixkokob | 20.951833 | -89.306194 | 0 | 3 | 1 |
|  | Tuzantán | 15.146703 | -92.422417 | 1 | 1 | 1 |
|  | Xcunyá | 21.133109 | -89.617642 | 1 | 0 | 0 |

**Supplementary Table S3** : Geographical distribution of the honey samples collected from honey bees. and stingless bees in Thailand (n=60) and Mexico (n=31)
